# 1-Benzoyl-3,6-diphenyl-1,4-dihydro-1,2,4,5-tetra­zine

**DOI:** 10.1107/S1600536810038067

**Published:** 2010-09-30

**Authors:** Feng Xu, Zhen-Zhen Yang, Xiao-Fang Chen, Wen-Xiu Zheng

**Affiliations:** a Department of Biological and Chemical Engineering, Taizhou Vocational and Technical college, Taizhou 318000, People’s Republic of China

## Abstract

In the title compound, C_21_H_16_N_4_O, the central tetra­zine ring adopts an unsymmetrical boat conformation with the two N atoms as the bow and stern. The crystal packing is stabilized by inter­molecular N—H—O hydrogen bonds.

## Related literature

For related structures, see: Xu *et al.* (2010 ▶); Hu *et al.* (2004 ▶); Rao *et al.* (2006 ▶). For applications of 1,2,4,5-tetra­zine derivatives, see: Sauer *et al.* (1996 ▶). 
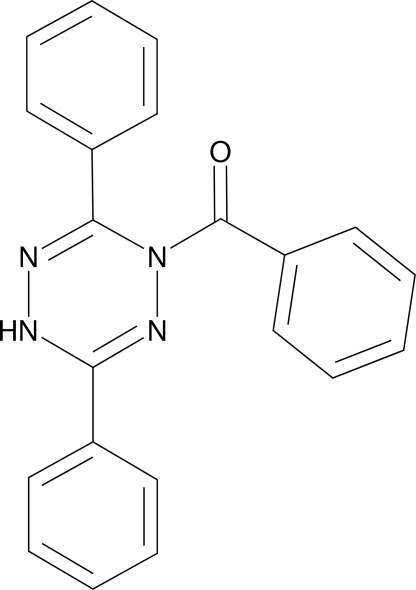


## Experimental

### 

#### Crystal data

C_21_H_16_N_4_O
*M* *_r_* = 340.38
Orthorhombic, 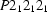

*a* = 7.1100 (19) Å
*b* = 12.115 (3) Å
*c* = 19.884 (6) Å
*V* = 1712.7 (8) Å^3^
*Z* = 4
Mo *K*α radiation
μ = 0.09 mm^−1^
*T* = 93 K
0.43 × 0.27 × 0.27 mm

#### Data collection

Rigaku AFC10/Saturn724+ diffractometer
13708 measured reflections
2254 independent reflections
2128 reflections with *I* > 2σ(*I*)
*R*_int_ = 0.032

#### Refinement

*R*[*F* ^2^ > 2σ(*F* ^2^)] = 0.033
*wR*(*F* ^2^) = 0.085
*S* = 1.00
2254 reflections
239 parameters
H atoms treated by a mixture of independent and constrained refinement
Δρ_max_ = 0.23 e Å^−3^
Δρ_min_ = −0.20 e Å^−3^

### 

Data collection: *CrystalClear* (Rigaku/MSC, 2008 ▶); cell refinement: *CrystalClear*; data reduction: *CrystalClear*; program(s) used to solve structure: *SHELXS97* (Sheldrick, 2008 ▶); program(s) used to refine structure: *SHELXL97* (Sheldrick, 2008 ▶); molecular graphics: *SHELXTL* (Sheldrick, 2008 ▶); software used to prepare material for publication: *SHELXL97*.

## Supplementary Material

Crystal structure: contains datablocks global, I. DOI: 10.1107/S1600536810038067/bq2236sup1.cif

Structure factors: contains datablocks I. DOI: 10.1107/S1600536810038067/bq2236Isup2.hkl

Additional supplementary materials:  crystallographic information; 3D view; checkCIF report

## Figures and Tables

**Table 1:** Hydrogen-bond geometry (Å, °)

*D*—H⋯*A*	*D*—H	H⋯*A*	*D*⋯*A*	*D*—H⋯*A*
N4—H4*N*⋯O1^i^	0.91 (2)	1.97 (2)	2.806 (2)	150.9 (18)

Symmetry code: (i) 
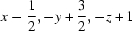
.

**Fig. 1.**
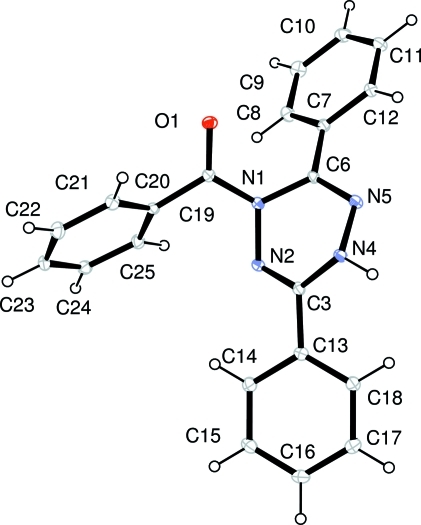
The molecular structure of the title compound, showing 30% probability displacement ellipsoids and the atomic numbering.

**Fig. 2.**
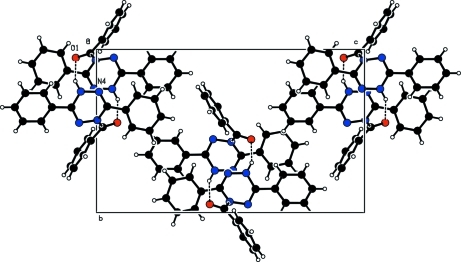
Crystal structure of (I) viewed down the a axis. N—H···O hydrogen bondings are shown as dashed lines.

## supplementary crystallographic information

### Comment

1,2,4,5-Tetrazine derivatives have high potential for biological activity, possessing a wide spectrum of antiviral and antitumor properties. They have been widely used in pesticides and herbicides (Sauer, 1996). Dihydro-1,2,4,5- tetrazine has four isomers, namely 1,2-, 1,4-, 1,6- and 3,6-dihydro-1,2,4,5- tetrazines. The 1,4-dihydro structures (Rao *et al.*, 2006) were found to have potential antitumor properties. In continuation of our work on the structure-activity relationship of dihydro-1,2,4,5-tetrazine derivatives (Hu *et al.*, 2004 & Xu *et al.*, 2010), we report here the crystal structure of the title compound (I) (Fig. 1).

In the tetrazine ring, atoms N2, C3, N5 and C6 are almost coplanar, while atoms N1 and N4 deviate from the plane by 0.447 (2) and 0.330 (2) Å, respectively. The N1/N2/C6 and C3/N4/N5 planes make dihedral angles of 35.76 (2)° and 27.66 (2)°, respectively, with the N2/C3/N5/C6 plane, *i.e.* the tetrazine ring adopts an unsymmetrical boat conformation. The benzene rings C7—C12, C13—C18 and C20—C25 make dihedral angles of 22.88 (2)°, 17.80 (2)° and 87.09 (2)° with the N2/C3/N5/C6 plane, respectively. Atom N1 is almost *sp*^2^ hybridized due to the angles around it add up to 358.0 (2)°.

The crystal packing (Fig.2) is stabilized by intermolecular N—H—O interactions between a hydrogen on nitrogen N(4) and the O atom of carbonyl group, with a N—H—O separation of 2.806 (2) Å (Table 2).

### Experimental

To a solution of 1,2-dihydro-1,2,4,5-tetrazine (0.47 g, 2 mmol) in chloroform (20 ml) was added dropwise benzoyl chloride (0.28 g, 2 mmol) in chloroform (10 ml) under stirring at room temperature using pyridine (0.17 g, 2.1 mmol) as the catalyst. After stirring for 2 h, the solvent was distilled off under vacuum. The residue was chromatographed on a silica gel column using cyclohexane-dichloromethane (V/V, 1:2) as the eluent to get the yellow solid (0.37 g, 55%). An anhydrous ethanol solution of the title compound was stood at room temperature, and by slowly evaporating ethanol from the solution, yellow crystals suitable for X-ray diffraction analysis were isolated one month later. m.p.: 500–501 K.

### Refinement

H atoms were placed in calculated positions with N—H = 0.86 Å, C—H = 0.93 (aromatic) and 0.96 Å (methyl), and refined in riding model, with *U*_iso_(H) = 1.2*U*_eq_(C,*N*) and 1.5*U*_eq_(C_methyl_). 1662 Friedel pairs were averaged before the final refinement as the absolute structure could not be determined unambiguously.

### Crystal data

**Table:**

C_21_H_16_N_4_O	*F*(000) = 712
*M**_r_* = 340.38	*D*_x_ = 1.320 Mg m^−^^3^
Orthorhombic, *P*2_1_2_1_2_1_	Mo *K*α radiation, λ = 0.71073 Å
Hall symbol: P 2ac 2ab	Cell parameters from 2254 reflections
*a* = 7.1100 (19) Å	θ = 3.4–27.5°
*b* = 12.115 (3) Å	µ = 0.09 mm^−^^1^
*c* = 19.884 (6) Å	*T* = 93 K
*V* = 1712.7 (8) Å^3^	Column, yellow
*Z* = 4	0.43 × 0.27 × 0.27 mm

### Data collection

**Table:**

Rigaku AFC10/Saturn724+ diffractometer	2128 reflections with *I* > 2σ(*I*)
Radiation source: Rotating Anode	*R*_int_ = 0.032
graphite	θ_max_ = 27.5°, θ_min_ = 3.0°
Detector resolution: 28.5714 pixels mm^-1^	*h* = −9→9
φ and ω scans	*k* = −15→14
13708 measured reflections	*l* = −24→25
2254 independent reflections	

### Refinement

**Table:**

Refinement on *F*^2^	Primary atom site location: structure-invariant direct methods
Least-squares matrix: full	Secondary atom site location: difference Fourier map
*R*[*F*^2^ > 2σ(*F*^2^)] = 0.033	Hydrogen site location: inferred from neighbouring sites
*wR*(*F*^2^) = 0.085	H atoms treated by a mixture of independent and constrained refinement
*S* = 1.00	*w* = 1/[σ^2^(*F*_o_^2^) + (0.0577*P*)^2^ + 0.196*P*] where *P* = (*F*_o_^2^ + 2*F*_c_^2^)/3
2254 reflections	(Δ/σ)_max_ = 0.001
239 parameters	Δρ_max_ = 0.23 e Å^−^^3^
0 restraints	Δρ_min_ = −0.19 e Å^−^^3^

### Special details

**Table:**

Geometry. All e.s.d.'s (except the e.s.d. in the dihedral angle between two l.s. planes) are estimated using the full covariance matrix. The cell e.s.d.'s are taken into account individually in the estimation of e.s.d.'s in distances, angles and torsion angles; correlations between e.s.d.'s in cell parameters are only used when they are defined by crystal symmetry. An approximate (isotropic) treatment of cell e.s.d.'s is used for estimating e.s.d.'s involving l.s. planes.
Refinement. Refinement of *F*^2^ against ALL reflections. The weighted *R*-factor *wR* and goodness of fit *S* are based on *F*^2^, conventional *R*-factors *R* are based on *F*, with *F* set to zero for negative *F*^2^. The threshold expression of *F*^2^ > σ(*F*^2^) is used only for calculating *R*-factors(gt) *etc*. and is not relevant to the choice of reflections for refinement. *R*-factors based on *F*^2^ are statistically about twice as large as those based on *F*, and *R*- factors based on ALL data will be even larger.

### Fractional atomic coordinates and isotropic or equivalent isotropic displacement parameters (Å^2^)

**Table:**

	*x*	*y*	*z*	*U*_iso_*/*U*_eq_	
O1	0.41566 (18)	0.55133 (9)	0.57735 (6)	0.0169 (3)	
N1	0.1772 (2)	0.56642 (12)	0.50300 (7)	0.0160 (3)	
N2	0.1136 (2)	0.55047 (12)	0.43512 (7)	0.0166 (3)	
N4	0.0860 (2)	0.74205 (12)	0.44154 (7)	0.0166 (3)	
N5	0.0366 (2)	0.74009 (12)	0.50949 (7)	0.0171 (3)	
C3	0.0818 (2)	0.64438 (14)	0.40646 (8)	0.0155 (3)	
C6	0.0824 (2)	0.65060 (13)	0.53963 (8)	0.0153 (3)	
C7	0.0230 (2)	0.62957 (14)	0.60945 (8)	0.0166 (4)	
C8	0.0051 (3)	0.52210 (15)	0.63299 (9)	0.0203 (4)	
H8	0.0357	0.4620	0.6043	0.024*	
C9	−0.0570 (3)	0.50186 (15)	0.69807 (9)	0.0237 (4)	
H9	−0.0689	0.4281	0.7137	0.028*	
C10	−0.1015 (3)	0.58866 (16)	0.74016 (9)	0.0234 (4)	
H10	−0.1438	0.5747	0.7847	0.028*	
C11	−0.0842 (3)	0.69637 (16)	0.71726 (9)	0.0230 (4)	
H11	−0.1144	0.7561	0.7463	0.028*	
C12	−0.0230 (3)	0.71750 (15)	0.65229 (9)	0.0197 (4)	
H12	−0.0124	0.7914	0.6368	0.024*	
C13	0.0390 (2)	0.64618 (14)	0.33354 (8)	0.0163 (3)	
C14	0.1052 (3)	0.55940 (15)	0.29386 (9)	0.0203 (4)	
H14	0.1729	0.5004	0.3140	0.024*	
C15	0.0725 (3)	0.55907 (16)	0.22504 (9)	0.0225 (4)	
H15	0.1169	0.4996	0.1982	0.027*	
C16	−0.0250 (3)	0.64547 (16)	0.19537 (9)	0.0217 (4)	
H16	−0.0468	0.6453	0.1482	0.026*	
C17	−0.0907 (3)	0.73210 (16)	0.23452 (9)	0.0234 (4)	
H17	−0.1570	0.7914	0.2141	0.028*	
C18	−0.0594 (3)	0.73225 (15)	0.30386 (9)	0.0200 (4)	
H18	−0.1054	0.7913	0.3307	0.024*	
C19	0.3449 (2)	0.52356 (13)	0.52322 (8)	0.0141 (3)	
C20	0.4384 (2)	0.44072 (14)	0.47866 (8)	0.0153 (3)	
C21	0.6317 (3)	0.45175 (15)	0.46805 (9)	0.0197 (4)	
H21	0.6976	0.5123	0.4872	0.024*	
C22	0.7276 (3)	0.37442 (15)	0.42963 (10)	0.0242 (4)	
H22	0.8583	0.3833	0.4211	0.029*	
C23	0.6319 (3)	0.28419 (16)	0.40369 (9)	0.0221 (4)	
H23	0.6978	0.2302	0.3782	0.027*	
C24	0.4404 (3)	0.27244 (15)	0.41477 (8)	0.0207 (4)	
H24	0.3757	0.2104	0.3969	0.025*	
C25	0.3424 (3)	0.35102 (14)	0.45181 (9)	0.0187 (4)	
H25	0.2108	0.3435	0.4587	0.022*	
H4N	0.048 (3)	0.8053 (17)	0.4209 (10)	0.020 (5)*	

### Atomic displacement parameters (Å^2^)

**Table:**

	*U*^11^	*U*^22^	*U*^33^	*U*^12^	*U*^13^	*U*^23^
O1	0.0209 (6)	0.0155 (6)	0.0144 (5)	−0.0013 (5)	−0.0018 (5)	0.0010 (4)
N1	0.0183 (7)	0.0151 (7)	0.0145 (6)	0.0020 (6)	−0.0008 (6)	−0.0025 (6)
N2	0.0181 (7)	0.0185 (7)	0.0132 (6)	0.0027 (6)	−0.0010 (6)	−0.0031 (6)
N4	0.0226 (7)	0.0142 (7)	0.0129 (6)	0.0022 (6)	0.0006 (6)	−0.0005 (5)
N5	0.0198 (7)	0.0172 (7)	0.0145 (7)	0.0004 (6)	0.0018 (6)	−0.0028 (5)
C3	0.0140 (8)	0.0153 (8)	0.0171 (7)	0.0000 (7)	0.0003 (6)	−0.0029 (6)
C6	0.0164 (8)	0.0126 (8)	0.0170 (8)	0.0012 (7)	−0.0021 (6)	−0.0039 (6)
C7	0.0140 (8)	0.0185 (9)	0.0172 (8)	−0.0001 (7)	−0.0017 (6)	−0.0020 (7)
C8	0.0201 (9)	0.0174 (8)	0.0233 (8)	−0.0022 (7)	0.0023 (7)	−0.0025 (7)
C9	0.0226 (9)	0.0227 (10)	0.0256 (9)	−0.0035 (8)	0.0032 (8)	0.0035 (7)
C10	0.0214 (9)	0.0323 (10)	0.0165 (8)	−0.0013 (8)	0.0031 (7)	0.0020 (7)
C11	0.0239 (9)	0.0273 (10)	0.0179 (8)	0.0037 (8)	0.0005 (7)	−0.0052 (7)
C12	0.0231 (9)	0.0179 (8)	0.0181 (8)	0.0039 (8)	−0.0008 (7)	−0.0019 (7)
C13	0.0141 (8)	0.0184 (8)	0.0162 (7)	−0.0025 (7)	0.0005 (6)	−0.0009 (6)
C14	0.0222 (9)	0.0180 (9)	0.0206 (8)	0.0016 (7)	−0.0017 (7)	−0.0019 (7)
C15	0.0240 (9)	0.0241 (9)	0.0195 (8)	−0.0004 (9)	0.0015 (7)	−0.0060 (7)
C16	0.0200 (8)	0.0303 (10)	0.0148 (7)	−0.0042 (8)	−0.0006 (7)	−0.0006 (7)
C17	0.0235 (9)	0.0256 (10)	0.0210 (8)	0.0022 (8)	−0.0044 (7)	0.0016 (7)
C18	0.0197 (9)	0.0213 (9)	0.0191 (8)	0.0023 (8)	−0.0005 (7)	−0.0023 (7)
C19	0.0173 (8)	0.0108 (7)	0.0143 (7)	−0.0013 (7)	0.0017 (6)	0.0027 (6)
C20	0.0197 (8)	0.0141 (8)	0.0121 (7)	0.0015 (7)	−0.0004 (6)	0.0017 (6)
C21	0.0189 (9)	0.0183 (9)	0.0219 (8)	−0.0006 (7)	−0.0013 (7)	0.0003 (7)
C22	0.0180 (9)	0.0276 (10)	0.0269 (9)	0.0035 (8)	0.0017 (7)	−0.0001 (8)
C23	0.0254 (10)	0.0218 (9)	0.0192 (8)	0.0093 (8)	0.0009 (7)	−0.0007 (7)
C24	0.0262 (9)	0.0162 (8)	0.0197 (8)	0.0018 (8)	−0.0054 (7)	−0.0025 (7)
C25	0.0191 (8)	0.0162 (8)	0.0208 (8)	0.0008 (8)	−0.0011 (7)	−0.0008 (7)

### Geometric parameters (Å, °)

**Table:**

O1—C19	1.235 (2)	C13—C14	1.396 (2)
N1—C19	1.361 (2)	C14—C15	1.388 (2)
N1—C6	1.423 (2)	C14—H14	0.9500
N1—N2	1.4363 (19)	C15—C16	1.387 (3)
N2—C3	1.292 (2)	C15—H15	0.9500
N4—C3	1.374 (2)	C16—C17	1.388 (3)
N4—N5	1.3961 (19)	C16—H16	0.9500
N4—H4N	0.91 (2)	C17—C18	1.397 (2)
N5—C6	1.281 (2)	C17—H17	0.9500
C3—C13	1.482 (2)	C18—H18	0.9500
C6—C7	1.473 (2)	C19—C20	1.495 (2)
C7—C8	1.389 (3)	C20—C25	1.390 (2)
C7—C12	1.403 (2)	C20—C21	1.396 (2)
C8—C9	1.389 (3)	C21—C22	1.388 (3)
C8—H8	0.9500	C21—H21	0.9500
C9—C10	1.381 (3)	C22—C23	1.387 (3)
C9—H9	0.9500	C22—H22	0.9500
C10—C11	1.388 (3)	C23—C24	1.387 (3)
C10—H10	0.9500	C23—H23	0.9500
C11—C12	1.387 (2)	C24—C25	1.391 (3)
C11—H11	0.9500	C24—H24	0.9500
C12—H12	0.9500	C25—H25	0.9500
C13—C18	1.387 (2)		
			
C19—N1—C6	122.48 (14)	C15—C14—H14	119.9
C19—N1—N2	120.12 (13)	C13—C14—H14	119.9
C6—N1—N2	115.36 (14)	C16—C15—C14	120.06 (17)
C3—N2—N1	110.55 (13)	C16—C15—H15	120.0
C3—N4—N5	118.11 (14)	C14—C15—H15	120.0
C3—N4—H4N	119.3 (13)	C15—C16—C17	120.02 (16)
N5—N4—H4N	112.0 (13)	C15—C16—H16	120.0
C6—N5—N4	113.79 (14)	C17—C16—H16	120.0
N2—C3—N4	122.04 (14)	C16—C17—C18	120.08 (18)
N2—C3—C13	118.71 (15)	C16—C17—H17	120.0
N4—C3—C13	119.25 (15)	C18—C17—H17	120.0
N5—C6—N1	119.17 (15)	C13—C18—C17	119.95 (17)
N5—C6—C7	120.97 (15)	C13—C18—H18	120.0
N1—C6—C7	119.61 (15)	C17—C18—H18	120.0
C8—C7—C12	119.05 (16)	O1—C19—N1	120.69 (15)
C8—C7—C6	120.39 (15)	O1—C19—C20	121.22 (15)
C12—C7—C6	120.52 (15)	N1—C19—C20	118.08 (14)
C9—C8—C7	120.56 (17)	C25—C20—C21	120.00 (17)
C9—C8—H8	119.7	C25—C20—C19	122.27 (16)
C7—C8—H8	119.7	C21—C20—C19	117.59 (16)
C10—C9—C8	120.20 (17)	C22—C21—C20	120.15 (18)
C10—C9—H9	119.9	C22—C21—H21	119.9
C8—C9—H9	119.9	C20—C21—H21	119.9
C9—C10—C11	119.80 (16)	C23—C22—C21	119.68 (18)
C9—C10—H10	120.1	C23—C22—H22	120.2
C11—C10—H10	120.1	C21—C22—H22	120.2
C12—C11—C10	120.47 (17)	C24—C23—C22	120.24 (18)
C12—C11—H11	119.8	C24—C23—H23	119.9
C10—C11—H11	119.8	C22—C23—H23	119.9
C11—C12—C7	119.92 (17)	C23—C24—C25	120.37 (18)
C11—C12—H12	120.0	C23—C24—H24	119.8
C7—C12—H12	120.0	C25—C24—H24	119.8
C18—C13—C14	119.71 (15)	C20—C25—C24	119.52 (17)
C18—C13—C3	122.06 (15)	C20—C25—H25	120.2
C14—C13—C3	118.20 (16)	C24—C25—H25	120.2
C15—C14—C13	120.18 (17)		
			
C19—N1—N2—C3	−123.18 (17)	N2—C3—C13—C14	−25.1 (2)
C6—N1—N2—C3	41.0 (2)	N4—C3—C13—C14	155.04 (17)
C3—N4—N5—C6	33.1 (2)	C18—C13—C14—C15	−0.1 (3)
N1—N2—C3—N4	−8.0 (2)	C3—C13—C14—C15	−178.39 (17)
N1—N2—C3—C13	172.13 (14)	C13—C14—C15—C16	0.5 (3)
N5—N4—C3—N2	−30.0 (2)	C14—C15—C16—C17	−0.3 (3)
N5—N4—C3—C13	149.85 (15)	C15—C16—C17—C18	−0.2 (3)
N4—N5—C6—N1	0.9 (2)	C14—C13—C18—C17	−0.4 (3)
N4—N5—C6—C7	−173.36 (15)	C3—C13—C18—C17	177.78 (17)
C19—N1—C6—N5	124.94 (18)	C16—C17—C18—C13	0.6 (3)
N2—N1—C6—N5	−38.8 (2)	C6—N1—C19—O1	5.0 (2)
C19—N1—C6—C7	−60.7 (2)	N2—N1—C19—O1	168.06 (15)
N2—N1—C6—C7	135.50 (16)	C6—N1—C19—C20	−176.05 (15)
N5—C6—C7—C8	154.45 (17)	N2—N1—C19—C20	−13.0 (2)
N1—C6—C7—C8	−19.8 (2)	O1—C19—C20—C25	130.67 (18)
N5—C6—C7—C12	−23.1 (3)	N1—C19—C20—C25	−48.2 (2)
N1—C6—C7—C12	162.63 (17)	O1—C19—C20—C21	−45.1 (2)
C12—C7—C8—C9	−0.1 (3)	N1—C19—C20—C21	136.01 (17)
C6—C7—C8—C9	−177.77 (17)	C25—C20—C21—C22	1.2 (3)
C7—C8—C9—C10	−0.1 (3)	C19—C20—C21—C22	177.08 (16)
C8—C9—C10—C11	0.1 (3)	C20—C21—C22—C23	−2.1 (3)
C9—C10—C11—C12	0.2 (3)	C21—C22—C23—C24	1.4 (3)
C10—C11—C12—C7	−0.5 (3)	C22—C23—C24—C25	0.1 (3)
C8—C7—C12—C11	0.4 (3)	C21—C20—C25—C24	0.3 (3)
C6—C7—C12—C11	178.06 (17)	C19—C20—C25—C24	−175.30 (15)
N2—C3—C13—C18	156.66 (17)	C23—C24—C25—C20	−1.0 (3)
N4—C3—C13—C18	−23.2 (2)		

### Hydrogen-bond geometry (Å, °)

**Table:**

*D*—H···*A*	*D*—H	H···*A*	*D*···*A*	*D*—H···*A*
N4—H4N···O1^i^	0.91 (2)	1.97 (2)	2.806 (2)	150.9 (18)

Symmetry codes: (i) *x*−1/2, −*y*+3/2, −*z*+1.
